# Facebook as an integrated online learning support application during the COVID19 pandemic: Thai university students’ experiences and perspectives

**DOI:** 10.1016/j.heliyon.2021.e08317

**Published:** 2021-11-03

**Authors:** Mark B. Ulla, William F. Perales

**Affiliations:** Walailak University, Thailand

**Keywords:** Connectivism, COVID19, e-learning, Facebook, Remote teaching

## Abstract

While a number of studies in the literature have explored the potentials of using Facebook in classroom teaching, there is a scarcity of studies that investigate its use as an online learning support application, especially when classes have to be moved to online and remote teaching due to health emergencies like the COVID19 pandemic. This article explores the use of a closed-class Facebook group (FBG) as a learning support application from the perspectives and experiences of 33 university English language students in Thailand. A self-report survey questionnaire and a semi-structured individual interview were conducted to get the data for the study. Drawing on the concept of connectivism, findings revealed that although students faced some issues like internet connectivity, lesson concentration difficulty, and lack of support from the family, they still held positive perceptions in moving to online class on Facebook during the pandemic. They believed that Facebook provided them with an easy way to connect with their classmates, who can support them in their remote language learning. Thus, Facebook is not only perceived as a social network by the students, but also as a learning platform where they can easily retrieve academic sources and share them with their classmates for intellectual discussion. This article argues that given the right online pedagogical strategy, Facebook can be used as an alternative to an established learning management system, especially when a university does not have one.

## 1 Introduction

The teaching and learning process of today has been admittedly influenced by the advancement of information and communication technology (ICT). In fact, using technology in the classroom and in teaching and learning online through distance and open education system (Almaghaslah et al., 2018; Bandalaria 2018; Mclsaac and Gunawardena 1996; Qayyum and Zawacki-Richter 2018) may have been commonly accepted as an effective means for teaching and learning in the 21st century. As a result, the relationship between ICT and pedagogy may have become a popular area of research by some education scholars who may be interested in investigating the benefits of integrating technology in classroom pedagogies. Previous studies (Barrot 2016; Sánchez et al., 2019; Ulla and Perales, 2020; Ulla et al., 2020) agreed that the presence of the internet and technology plays a significant role in language education. Such presence of different technologies in language education has transformed the classroom's pedagogical landscape and has offered an opportunity for both teachers and students to have easy access to learning connections and rich information online. For instance, teachers can now access and download prepared lesson plans and research studies that they could use to inform their teaching practices. They can also connect to other teachers, forming a community of practice (CoP) that is crucial for knowledge creation and dissemination (Wesely, 2013). Likewise, students can now form their language learning networks and communities with their classmates and peers for support and or to facilitate and be involved in the process of continuous learning.

In higher education institutions (HEIs), using a proper learning management system (LMS) may be considered as one of the most important developments of technology, especially in managing the teaching and learning system. An LMS is a system that provides teachers and students a safe and secured venue for online teaching and learning (Aldiab et al., 2019). It manages and monitors students’ learning progress, provides a communication link between students and teachers, and serves as a platform for assessment, testing, and evaluation (Vicheanpanya 2014). Utilizing an LMS is crucial for improving an online and remote teaching and learning process since it provides teachers and students a platform to connect academically. Thus, considering the role of an LMS in online and remote teaching, its use during the COVID19 pandemic may be deemed necessary.

Generally, the rapid transmission of the coronavirus disease (COVID19) in late 2019 has negatively affected all aspects of society including education. A number of schools in the world migrated from face-to-face classroom teaching to online teaching. Schools and universities have had to find alternative ways by exploring possible online teaching platforms and strategies to continue the teaching and learning process. However, the lack of teacher's online pedagogical skills, the availability of electronic devices, and other technical issues (e.g., internet connectivity, electricity, etc.) (Farley and Song 2015; Nhu et al., 2019), and the availability of an online teaching platform remained to be the main issues encountered by a number of teachers around the world. While there may be a number of schools that may have an established online Learning Management System (LMS) (Han and Shin, 2016), other schools may only employ social media platforms for their online teaching (Chawinga 2017).

In Thailand, the COVID19 pandemic has also impacted the teaching and learning process in all schools across the country. As a result, face-to-face classroom teaching had to stop temporarily while other schools had to migrate to online and remote teaching. Thus, online platforms such as *Zoom, Microsoft Teams*, and *Google classroom* may have become popular not because they offer convenience for both teachers and students but because some schools may not have a proper LMS. In the author's university, using a common LMS was not clearly emphasized when moving from face-to-face classes to online and remote teaching during the COVID19 pandemic. Although the university had an LMS in place, the sudden spread of the coronavirus caught the university and all its teachers unprepared to utilize the existing LMS. As a result, teachers utilized various online platforms to continue the teaching and learning process.

Drawing on the concept of connectivism (Siemens, 2005), this article presents Thai university students’ perceptions and experiences of online learning using Facebook as an online learning support application during the COVID19 pandemic. It is hoped that the findings from this study will contribute to the growing literature on the use of social media as an online learning support for distance learning in times of health emergencies when classes are disrupted and when a proper LMS is not available.

## 2 Related literature

In the absence of an LMS, the availability and accessibility of various popular social media may be the only platforms that provide teachers with a convenient and easy way to navigate through online teaching. Social media platforms such as *Facebook*, *Twitter*, *LINE*, *Hangout*, and *WhatsApp* may offer a good alternative for classroom-based teaching as these platforms may also promote interaction among teachers and students. In fact, a large number of studies in the literature (Chugh and Ruhi 2018; Tang and Hew 2017; Wesely 2013) have proven that the use of these platforms is beneficial for both teachers and students. Although the use of these social media platforms is limited only to teaching a specific language skill, they still enhanced students' learning of the language (Ulla and Perales, 2020). For example, Hamat and Abu Hassan (2019) admitted that the use of social media in teaching the English language can greatly contribute to students' improvement in writing, communication, vocabulary development, and reading. Social media can also facilitate online interaction, discussion, and communication (Aydın and Özdemir 2019). In addition, social media platforms can also enhance vocabulary learning (Sato et al., 2013), promote learners' learning autonomy (Sato et al., 2015), and provide students' the opportunity to become responsible learners (Bezircilioğlu 2016). Lastly, the use of social media in English language teaching can make the students’ language learning experience fun, motivating, exciting, interesting, and engaging (Henry et al., 2020; Sánchez et al., 2019; Ulla and Perales, 2020).

Subsequently, Facebook is one of the most popular social media sites that is also integrated in ELT classroom. Such popularity motivates scholars and education practitioners to undertake a study investigating the potentials of Facebook as an online teaching and learning platform. For example, Ulla and Perales (2020) conducted a study where they used Facebook as a class white-board in English as a foreign language class. The results revealed that based on the students' engagement to various classroom tasks, Facebook as a class white-board can promote high students’ participation which can greatly improve effective language teaching and learning. However, it is also acknowledged that the use of Facebook in classroom teaching comes with some issues that have to be dealt with by education practitioners. For instance, the study conducted by Barrot (2016) examined the problems encountered by 171 first-year university students in the Philippines with regard to the use of Facebook as an e-portfolio platform. Using a self-report questionnaire, the study revealed that although students had a positive perception of the use of Facebook as an e-portfolio platform, they also reported some issues on format and readability. Some students reported that they could not read the texts posted on Facebook as they appeared to be relatively small. Others also complained about the formatting of their list of references on Facebook. Most importantly, students were also anxious to post their output on Facebook since they were afraid to be criticized.

Similarly, the study conducted by Camus et al. (2016), which explored and compared the effects of using Facebook between the university-sponsored LMS to students’ participation, performance, and engagement to class activities, found that “while Facebook may be better at fostering student participation and encouraging peer-to-peer dialogue, the university-sponsored LMS may be a more effective tool for encouraging students to develop coherent arguments and apply course content in other contexts” (p. 84).

The findings from Barrot (2016), Camus et al. (2016), and Ulla and Perales (2020), although they contribute to the discussion on the use of Facebook in language classrooms, these studies focused only on the viability of the said platform as an integrated ICT into the language classroom. In other words, the findings did not point to how Facebook is used when there is no university-sponsored LMS and how it is used especially in the context of distance education during the COVID19 pandemic.

### 2.1 Facebook as a learning platform

Using social media in teaching and learning during the COVID19 pandemic may offer a good alternative to face-to-face classroom teaching as it supports active learning among students (Greenhow and Chapman 2020). However, while there are a number of studies in the literature (Barrot 2016; Camus et al., 2016; Henry et al., 2020; Sánchez et al., 2019; Ulla and Perales, 2020) that reported the educational benefits of using social media in classroom teaching, there is a scarcity of studies that investigate the use of Facebook as an online learning support application especially when a university does not have an established LMS and when classes have to be moved to online and remote teaching due to health emergencies. In fact, Niu (2017), in her review on the use of Facebook for academic purposes found that although Facebook has been widely recognized as a learning platform, which promotes student-centered learning, “the effects of Facebook used as learning management system are mixed and under-studied” (p. 1384). Such findings have been concurred with the study conducted by Barrot (2018) on using Facebook as a language learning environment. Barrot's literature review found that research studies that examined Facebook's role in language learning conducted in most Asian HEIs focused only on the development and the improvement of general language skills. In other words, studies that concentrate on using Facebook as an online learning support application are scant.

Contrary to the findings presented by Barrot (2018) and Niu (2017), the review conducted by Manca and Ranieri (2016), although they mentioned that Facebook was mostly used as an alternative to LMS, admitted some learning designs differences in most of the reviewed studies. Such differences include posting of assignments, delivering content, and providing feedback. However, despite these differences, Facebook is still considered as an “informal, dynamic, social and flexible environment where more or less structured learning experiences can take place” (p. 520). Similarly, Chugh and Ruhi (2018) also asserted that Facebook can be used as a platform for informal and formal language learning.

The studies mentioned above, while they made an important contribution in investigating the use of Facebook as an online learning support application, lacked empirical evidence as these studies only reviewed existing literature. Additionally, the studies that were reviewed were conducted not in times of emergency situations where classes are disrupted and have to be moved completely online. Thus, there is a need to investigate the use of Facebook as an online learning support application, especially in online and remote teaching and learning during the COVID19 pandemic. Empirical studies, such as the present study, is important to identify the viability of the said platform to be used fully in distance education. Furthermore, exploring students’ experiences in online learning and how learning takes place in this networked environment can deepen our understanding of the concept of distance learning during health crises.

## 3 Theoretical approach: connectivism as a learning theory

The term connectivism was introduced by George Siemens in 2005 as “a learning theory for the digital age” (Siemens 2005:1). As a learning theory, it emphasizes the role of technology in the learner's learning process. It asserts that technology plays a crucial part in the process of accessing information from various sources and the advancement of skills in an active information network (Dunaway 2011). Connectivism maintains that learning is a process of connecting various information sources (Siemens 2005) that allow learners to form a learning community. Additionally, connectivism's important features can be summarized as follows. One, it recognizes the position of various digital technologies and the internet towards the process of learning. Two, it posits that learning happens when learners make sense of the information, which they get from various information sources. Lastly, it acknowledges that learning is a connection process that lies within the diversity of opinions, fields, and concepts. In other words, connectivism offers a modern viewpoint about how learning takes place in interactive media environments (Dunaway 2011).

Goldie (2016) claimed that in connectivism, learning takes place when learners are connected to a learning network of similar interests, where it allows them to freely interact and share their ideas with other people in the network. Through a learning network, learners do not only participate in the exchange of ideas, which facilitates the creation of knowledge, they also become part of the network. Learners’ participation in the learning network “consists not only of words but also of images e.g. video, multimedia, etc.” (Goldie 2016:5) that stimulate the learning process. Thus, learning in the framework of connectivism occurred when there is a connection and interaction between teachers, students, and in a specified learning network.

Generally, connectivism, as a learning theory is especially important in education in the age of the internet and technology as it provides many education experts, practitioners, and policy-makers a framework that can inform classroom pedagogy and education policies. In fact, one of its principles that may have been applied by some higher education institutions is the concept of learning that resides from non-human appliances. These non-human appliances may in fact refer to computers and the internet that transform the pedagogical landscape and changes how knowledge is acquired.

Connectivism is especially important in the present day not only because of the availability of different technology but because technology enables teachers and learners to form a network of learning communities that help them to learn more and thus be involved in the creation of new knowledge. Through the concept of connectivism, it helps offers new perspectives on how learning takes place in digital learning spaces. In the context of open/distance education, for instance, connectivism allows both teachers and students to learn and acquire new knowledge outside of the classroom and enables them to connect to their peers which could facilitate continual learning.

## 4 Research problems

This study sought to answer the following research questions:

1. How do Thai university students perceive emergency online remote learning on Facebook?
2. How do Thai students make use of Facebook to connect academically with their classmates for continuous learning?

## 5 Methodology

The present study employed phenomenology (Groenewald, 2004) as an approach to explore the lived experiences of Thai university students as regards remote learning on Facebook during the COVID19 pandemic. Phenomenology is a qualitative method of research that aims to describe and understand a certain phenomenon as experienced by the participants (Groenewald, 2004). Additionally, it made use also of a researcher-made self-report survey questionnaire to support the data obtained from the semi-structured individual interview.

The study was approved by the Walailak University Human Research Ethics Committee (WUEC-21-085-01).

### 5.1 Participants and context

The study attempts to describe and understand the experiences of Thai university students, their perceptions of Facebook as a learning support application, the issues they encounter, and how they address those issues when learning online during the COVID19 pandemic.

There were 33 (24 females, 9 males) students, whose age ranged between 19 and 21 years old enrolled in one of the author's general English classes, answered the survey questionnaire and only nine students participated in the interview. All of them took Medicine as their major and they spoke Thai as their first and English as their foreign languages, respectively. Their English language proficiency level is Intermediate based on the university English language proficiency test. Informed consent was also obtained from all the participants. These students were informed about the purpose of the study and it was also made clear to them that their participation was voluntary; and that all the information they would share would be treated with the utmost confidentiality.

The study made use of convenience sampling (Farrokhi and Mahmoudi-Hamidabad, 2012) as these students belonged to one of the author's classes. Likewise, a survey questionnaire and a semi-structured individual interview either through a questionnaire or Facebook chat (Ditchfield and Meredith, 2018) were conducted to get the data for the study. To ensure the safety and security of the students' identity online, a closed-class Facebook was created (Ulla and Perales, 2020).

### 5.2 Research tools and data analysis

Although the present investigation followed the qualitative research design with phenomenology as the research approach, it also made use of a researcher-made self-report survey questionnaire. The self-report questionnaire allows the participants to directly answer questions about themselves and their beliefs, perceptions, experiences, feelings, and attitudes towards certain phenomena (Korb 2011). Considering the nature of the study, which followed the phenomenological tradition of qualitative research, researcher-made self-report survey questionnaire is deemed appropriate as a research tool for the study. In designing the questionnaire, the researchers brainstormed about the possible questions relevant to the research problems posed in the study. The questionnaire was checked by their colleagues for repetition, unclear statements, and leading questions. Furthermore, the survey questionnaire had 3 parts. Part one asked about the students' profiles. The second part dealt with their perceptions of online learning on Facebook. Part three asked about students’ issues and difficulties in online learning on Facebook. The questionnaire, which was written in English, consisted of 20 items on the 4-Likert scale.

Likewise, the semi-structured individual interview was done during the final week of the semester. Since the class was moved online, the interview was also done through a questionnaire and Facebook chats. In the interview, students were asked about how they make sense of their learning context for continuous learning and how they make sure that they are still connected with their classmates academically on Facebook during the pandemic. To maintain their anonymity, students were assigned codes (Student 1, Student 2) in the presentation of the interview excerpts.

Lastly, the data for the survey questionnaire was tabulated using frequency count and percentage. Likewise, using the thematic analysis approach (Braun and Clarke, 2006), interview transcripts were read many times and were arranged, coded, and assigned to different themes that reflect the purpose of the study. To ensure validity, student-participants received back the interview transcripts for correction, addition, and approval.

## 6 Findings

*A. How do Thai university students perceive emergency online remote learning on Facebook?*

Data from Table 1 presented that majority of the students expressed a positive perception of Facebook as their online learning platform during the pandemic. They agreed that Facebook learning made them independent (81%) and creative learners (67%), where they could freely explore their ways of learning the course (81%).

**Table 1:** Students’ perceptions towards moving classes online on Facebook.

Statements	Strongly agree	Agree	Strongly disagree	Disagree
1. I am more comfortable communicating with my teacher on Facebook.	19%	57%	3%	21%
2. I am more comfortable learning on Facebook with my classmates.	15%	54%	3%	28%
3. I prefer to work and learn on Facebook.	16%	47%	8%	29%
4. I believe that online learning on Facebook is more convenient than classroom learning.	11%	39%	16%	34%
5.I believe online learning on Facebook helps me understand some concepts of the course.	10%	46%	7%	37%
6. I believe that I can do many things when learning on Facebook.	19%	56%	2%	23%
7. I believe that online learning on Facebook is more effective than learning in the classroom.	8%	35%	18%	39%
8. I believe that online on Facebook learning makes me an independent learner.	20%	61%	3%	16%
9. I believe that online learning on Facebook makes me a creative learner.	13%	54%	5%	28%
10. I believe online learning on Facebook gives me freedom to explore my way of learning the course.	24%	57%	3%	16%

In the interview, they expressed that learning on Facebook not only offers them the convenience of learning but also it made them safe from contracting the virus. When asked why they thought Facebook was a better online learning platform, their common response was that it offers them accessibility and convenience. These can be reflected in the following excerpts:

Since the start of the term when there was no COVID19 yet, we have been on Facebook already. Our section has its own Facebook group so I think using it for our online learning means that I don't have to adjust to a new learning platform. And also, I feel safe learning at home. (Student 1)

Everybody has Facebook and it was not a problem moving to online learning because everybody knows the platform already. I believe that in Facebook, we are able to continue learning outside of the classroom. (Student 2)

In addition, majority of the students (54%) said that they are comfortable learning on Facebook because they had “no problem with the internet” and their “parents were there ready to support” and help them. Similarly, student 4 also expressed that she “preferred to work and learn online” because she can do many things at home. For other students, they also revealed that it was easier to learn on Facebook because they “can go back and review the lessons any time after the class” since all the “lessons and activities were just posted on the class Facebook group page”. However, although students felt comfortable communicating with their teacher on Facebook (76%), they disagreed that online learning on Facebook was more effective than learning in the classroom (57%). They also disagreed that online learning on Facebook helped them understand some concepts of the course (44%). Such disagreements were often linked to some technical issues that students faced during their online learning. However, they learned how to mitigate these issues to achieve successful learning. In the interview, students disclosed;

I do not have a strong internet at home but I always make sure that whenever I have an online class, I have with me my textbook so I can be guided. The good thing about online learning on Facebook is that we only used it as a discussion board and no video. I can go back to it anytime”. (Student 9)

For student 8, a noisy home environment was his issue when learning online. He, however, mentioned how he addressed that issue.

Our family is big so it is very noisy. But when I have a class online, I have to close the door of my room so no one can get in. I also let my family members know that I am having an online class. At first, they were not supportive but after talking to them why I had to do online learning, they understood it.

The lack of teacher and or classmates' support for online learning was student 7's problem when learning online. However, he mentioned:

The only issue I have with online learning is that sometimes it is very hard to find support from your teacher and from your classmates given the situation that we are all on the computer screen only. But, whenever I have important questions or clarifications, I emailed my teacher or chat my classmates. It works for me.

Lastly, for student 5 on the issue of homework, she addressed that issue by asking for help from her family. She said:

My mother always helps me with my homework. We have a lot of homework but I was able to do them with the help of my family.

All of the students, although they held a positive perception towards learning online specifically on Facebook, also encountered some issues and problems (See Table 2). Among these issues are internet connectivity (75%), noisy home environment (61%), lack of support from family (40%), difficulty understanding the lesson online (60%), and the increased number of homework (79%). These issues as revealed by the participants had a negative effect on their academic performance.

**Table 2:** Common issues that Thai university students encounter when learning online on Facebook.

Statements	Strongly agree	Agree	Strongly disagree	Disagree
1. I have a weak internet connection at home.	25%	50%	5%	20%
2. I do not have the technological skills to do online learning.	17%	29%	7%	47%
3. I do not have a computer/laptop/mobile phone to use for online learning.	8%	24%	32%	36%
4. I cannot concentrate in online learning due to noisy home environment.	18%	43%	10%	29%
5. I have difficulty understanding the lesson online.	11%	49%	7%	33%
6. There are so many homework when learning online.	37%	42%	4%	17%
7. There is no support from my family when learning online.	11%	29%	19%	41%
8. Learning online is causing me stress and anxiety.	23%	43%	8%	26%
9. Learning online is lonely and sad.	20%	43%	9%	28%
10. Learning online is difficult.	16%	49%	5%	30%

*B. How do Thai students make use of Facebook to connect academically with their classmates for continuous learning?*

Several features of Facebook were reported to be useful during the online learning. During the interview, although students acknowledged that it was difficult for them to organize and gather together online to discuss some of the class activities, they managed to form their network to help and learn from each other in their class activities with the help of these features. For example, the group feature of Facebook becomes their exclusive community, connecting them with their teacher and their classmates. The chat function provides a place where students can discuss their online tasks. The discussion forum wall and the comment section also allow them to exchange academic ideas with their peers.

Forming a group for a class requirement online is a challenge but I learned to connect with my classmates easily. I chat my classmates on Facebook messenger and we discuss our homework and other class activities. (Student 1)

Unlike any LMS, Facebook provided the students with a convenient venue not only to reconnect with one another socially but also academically. As evident in the transcripts, engagement, and participation on Facebook online discussion forum were highlighted by the students. Through discussion forums, students shared their ideas with the members of the class.

I see Facebook as a convenient way to talk about homework with my classmates. So funny because we only used to chat and gossip on Facebook but now, we used it for our class and I like it so much because I got to share my ideas with them in their post. (Student 2)

Students 4 and 5 supported student 2's statement when they said that:

For me, I learn better when I participate in the discussion forum on Facebook. That is why I always comment on the post of my classmates because I also got ideas from them.

Personally, I like using Facebook for online learning because I can easily see the work of my classmates. And I learn more from them.

Pedagogically, online learning on Facebook has also brought positive effects to the students. They admitted that the they become responsible language learners.

Honestly, I can say that I become a responsible student. Even if I did not have to do the classwork with my classmates physically, but I make sure that I contribute something for the group and for the class to learn. (Student 6)

Being creative and resourceful language learners was also emphasized by students 4 and 8.

They said:

I become a creative person. When our teacher gave us video homework, I made sure that it was beautifully made so I can get a good grade”. (Student 4)

For me, I think, I become a resourceful student because when our student posted something about homework and other activities, I searched and looked for other references that I can read to supplement what my teacher was saying. (Student 8)

## 7 Discussion: students perceptions on online remote learning on facebook

The present study explored Thai university students’ perceptions and experiences in attending emergency remote and online learning classes on Facebook during the COVID19 pandemic in Thailand. Results from the survey questionnaire and the semi-structured individual interview revealed that although students encountered some issues in online learning during the pandemic (e.g., lack of support, interaction, concentration, internet connection, and more homework), they still held positive perceptions with regard to moving classes online. For example, despite the sudden transition to online learning and the lack of a proper learning management system (LMS), student-participants agreed that moving to an online class on Facebook in the middle of the semester, especially during the COVID19 pandemic, was a good decision. They believed that moving to an online class would be advantageous for them for some reasons. First, it would prevent them from contracting the virus. Second, they feel safe at home, especially that they were with their families. Third, it offered them a space for continuous learning since they are already familiar with the platform. Lastly, Facebook provides them a chance to become independent, responsible, creative, and resourceful learners as they got to work on the online activities either individually or collaboratively. Although of different contexts and participants, this finding is in contrast with the study conducted by Dong et al. (2020) in which they reported that parents had a negative attitude towards online learning during the COVID19 pandemic. Such negative beliefs on online learning were linked to the lack of time and professional knowledge among parents to assist their children for online learning.

However, it should be remembered that online learning is a new experience for the students, especially in the middle of a pandemic. They may not be prepared for the sudden transition to online learning, and they may find it difficult to adjust to a new learning environment. Nevertheless, it is evident that students positively received the transition to online learning from face-to-face classroom teaching. This can be attributed to the fact that students are technologically savvy, where they “routinely use digital playback devices in their lives for entertainment and communication to the point that students being “plugged in” is a ubiquitous image” (Gray et al., 2017, p. 28). Thus, it is not surprising how they adapted to the situation where they have to learn remotely.

Furthermore, working on some online activities alone may also be something that is not common, especially in a face-to-face classroom teaching and learning. Studies conducted by Hung and Mai (2020), Wilson et al. (2017), and Zubiri-Esnaola et al. (2020) emphasized that students may be put in groups or in pairs to do some activities for more classroom interaction and participation in a residential classroom teaching. However, this kind of classroom arrangement may be difficult when done in online remote teaching. Conducting online collaborative tasks and other group work may be a challenging task among teachers and students. Consequently, students may have to rely only on themselves to accomplish different online learning tasks. Additionally, while group work in face-to-face teaching may encourage collaborative learning and a learner-centered classroom (Hung and Mai 2020; Lau and Jin 2019), it should be considered that doing remote online activities alone may also bring a positive impact on student's language learning. Not only does it enhance the skills of the students to become independent learners, but it also offers them space where they can be resourceful and creative language learners.

It must be noted that even before the pandemic, the use of Facebook in classroom teaching has already been a popular area of research. In fact, the studies conducted by Camus et al. (2016) and Ulla and Perales (2020) revealed that students were more engaged, participative, and creative in doing classroom activities. It may also improve students' classroom performance (Sánchez et al., 2019), develop students’ communicative skills (Aydın and Özdemir 2019), and provide a place where students can connect with their peers for learning support (Pai et al., 2017). However, while the mentioned studies explored the use of Facebook in their respective contexts, none of these studies investigated how such a social media platform can be fully utilized in online and remote teaching. In the absence of an LMS and in times of health and socio-political crisis when classes are disrupted indefinitely, the use of social media platforms such as Facebook may provide a better alternative to face-to-face classroom teaching. However, while this study presents an online pedagogy on Facebook, this does not mean that this can also be effective in some other contexts. Considering some factors like internet connection and the availability of electronic devices, using Facebook in remote teaching still largely depends on how the teacher adapts the lesson online and on what teaching method is used. In other words, a good lesson plan is still important in online pedagogy.

### 7.1 Facebook as an academic platform

Another important finding from the study is that Facebook is not only perceived as a social network by the students. They also considered it as a learning platform where they can retrieve academic sources easily and share them with their classmates for intellectual discussion. Such finding has also been reported by Zarzour et al. (2020) in their study, which found that “the social features [of Facebook] including post, like, comment and share, were the main behaviors identified during the learning activity [among the students]” (p. 7). By posting discussion questions on Facebook, students can express their academic opinions and participate in creating new knowledge. It can stimulate critical thinking and allow students to interact, share, dialogue, and think together (Siemens 2005). Similar to the study conducted by Jogezai et al. (2021), social media, like Facebook, can be used for online instruction during the COVID19 pandemic. In this study, we argue that not only Facebook provides a space for academic discussion, it also serves as a platform for students to form an academic network where they can draw support and learn from each other. Given that most of the students are on Facebook, and thus, familiar with the platform, students develop the skills and strategies to continue learning and facilitate the creation and consumption of knowledge. This finding corroborates the idea of connectivism expressed by Kop and Hill (2008) that:

People can move from a learning environment controlled by the tutor and the institution, to an environment where they direct their own learning, find their own information, and create knowledge by engaging in networks away from the formal setting. They still communicate with others, but their personal interests and preferences – rather than institutional requirements and choices – are the main drivers for their engagement with more knowledgeable others in their learning (p. 9).

Learning in this connected network happened when students are engaged in the discussion and participated in the online class activities, which are different from traditional face-to-face classroom learning. Facebook as an online learning support also promotes language learning as it caters to different learning preferences among students. “Although orientation and training are necessary for learners to reach out, the quality of learning, peer support and supervision provides the true nature of the teaching and learning process” (Sozudogru et al., 2019, p. 356). In other words, when students are provided with a supportive, collaborative, and reflective environment, they may be able to “produce knowledge and increase time management, reflection, negotiation and communication skills” (Sozudogru et al., 2019, p. 356).

Furthermore, students’ participation and engagement in different online class activities enable them to become responsible for their learning. When they engage in an online discussion, they control their learning since they choose whom to engage with. This implies that students have to identify their source of knowledge and learn from it to share with the rest of the class. In other words, students become “the center of the learning experience, rather than the tutor and the institution. Learners will be instrumental in determining the content of the learning, in addition to deciding the nature and levels of communication, and who can participate” (Kop and Hill 2008: 9). Thus, using Facebook as an online learning support application during the COVID19 pandemic offers “benefits for active learning, community-building and civic participation that can help reduce some of the distance students feel” (Greenhow and Chapman 2020:348).

Theoretically, the study contributes to the existing literature on the use of Facebook as an online learning support application in that it presents empirical evidence on Thai students’ perceptions of Facebook during the pandemic. While studies mentioned in the literature only dealt with the integration of Facebook in classroom teaching, the present study fully employed the said online platform as a teaching and learning application during the pandemic. Practically, the present study confirmed that the use of Facebook is not only limited to being a social media platform, it can also be an effective teaching and learning support platform, especially during a pandemic.

However, while the present study offers a different perspective on online learning on Facebook in times of the pandemic, some limitations are also acknowledged. First, the present study only includes one group of students in a university English language class in Thailand. Future studies can include a bigger sample from different classes in the country to further explore Facebook as an online learning support application. Second, the study employs the conceptual framework of connectivism to identify how learning is done on Facebook. Using other frameworks may also bring a different perspective on using Facebook for online learning. Finally, the study concentrates only on using Facebook as an online learning support application during the COVID19 pandemic. Comparing the viability of Facebook as an online learning support application with other university-sponsored LMS may also be a good study to undertake.

## 8 Conclusion

The unprecedented COVID19 outbreak has indeed impacted the teaching and learning process. From the traditional face-to-face classroom teaching, a number of schools in the world have had to migrate to online and remote teaching in order not to disrupt the classes. With this health crisis, it makes us realize how important it is to have an online learning management system (LMS) that can be used when future disruptions of classes are inevitable. Although having a clear and established LMS may be of great help when transitioning from face-to-face classes to online classes, it cannot be denied that there may be a number of schools and universities in the world that do to have an LMS or to have one may be difficult due to financial constraints. Thus, the use of various social media platforms can become an alternative mode of delivering classes online. Therefore, in times of emergency and in health crisis where it affects the classroom teaching, the use of these online applications and platforms should be considered pedagogically.

In addition, when the threat of the COVID19 would be gone soon, education institutions all over the world would no longer be back to what we called the “old normal”. Instead, the “new normal” would become a conceptual practice that reminds all educators to adapt to the new reality in education where “the application of e-learning [education] is poised to become much more prominent” (Pham and Hanh Ho 2020:1). To cope with the demands of the “new normal” in education, teachers should update their skills and knowledge on the use of various information and communication technology tools in teaching. Technology integration in the classroom may no longer be optional but must be required so that classes will not be disrupted when other health and socio-political crisis may affect the delivery of the lesson in the future. The government, through its education department, should also be able to support these teachers by providing them technological training and technological support. Sahlberg (2020) mentioned that “hopefully, when this crisis is over politicians decide to continue to follow that same strategy and use more professional wisdom and evidence from education professionals to inform the next education policies and school reforms” (p.5-6). In other words, the government should continue giving its full support to teachers even when this pandemic is over. Lastly, the COVID19 also sees the need for the reform of the education curriculum; that is, the course syllabus should include and adapt various teaching strategies and other teaching online platforms that can be used in class. Thus, with this pandemic, various lessons and realization with regard to classroom teaching have been seen and considered. These lessons should be learned so that teachers and other education scholars are better prepared in times of crisis.

## Declarations

### Author contribution statement

Mark Ulla: Conceived and designed the experiments; Analyzed and interpreted the data; Wrote the paper.

William Perales: Performed the experiments; Contributed reagents, materials, analysis tools or data.

### Funding statement

This research did not receive any specific grant from funding agencies in the public, commercial, or not-for-profit sectors.

### Data availability statement

No data was used for the research described in the article.

### Declaration of interests statement

The authors declare no conflict of interest.

### Additional information

No additional information is available for this paper.
